# Different mutational characteristics of TSG in cell lines and surgical specimens

**DOI:** 10.1007/s13277-014-2444-5

**Published:** 2014-08-14

**Authors:** Ewelina Stoczynska-Fidelus, Michal Bienkowski, Marcin Pacholczyk, Marta Winiecka-Klimek, Mateusz Banaszczyk, Jolanta Zieba, Grzegorz Bieniek, Sylwester Piaskowski, Piotr Rieske

**Affiliations:** 1 Department of Tumor Biology, Medical University of Lodz, Lodz, Poland; 2 Department of Molecular Pathology and Neuropathology, Medical University of Lodz, Lodz, Poland; 3 Institute of Automatic Control, Silesian University of Technology, Gliwice, Poland; 4 Department of Orthopedics and Traumatology, Pabianice Medical Center Ltd., Pabianice, Poland

**Keywords:** Tumor suppressor genes, Quasi-sufficiency, Three hits hypothesis, Two hits hypothesis

## Abstract

One of the most crucial concerns of cancer research pertains to the differences between the neoplastic cells in tumor specimens in vivo and their counterparts in cell lines. The huge amount of results deposited in cancer genetic databases allows to address this issue from a wider perspective. Our analysis of the Sanger Institute Catalog Of Somatic Mutations In Cancer (COSMIC) database v61 showed a lower percentage of homozygous mutations in a group of tumor suppressor genes in surgical samples (in vivo) in comparison to their frequency in cell lines (in vitro). Similarly, the mutations resulting in the lack of protein (e.g., nonsense mutations or whole gene deletions) of several tumor suppressor genes (TSGs) were more frequently observed in vitro than in vivo. In this article, we suggest two potential explanations of these data. Firstly, TSG heterozygous mutations resulting in the modified protein (e.g., missense mutations) may be gradually (when the specific molecular context is achieved) changed to homozygous mutations resulting in the lack of protein during carcinogenesis. Secondly, among different independent pathways of tumorigenesis, those leading to homozygous nonsense mutations are characteristic for cells which are more efficiently stabilized in vitro. To conclude, these observations may be interesting for researchers working with cell line in vitro models illustrating the extent to which they reflect the tumors in vivo.

**Electronic supplementary material:**

The online version of this article (doi:10.1007/s13277-014-2444-5) contains supplementary material, which is available to authorized users.

## Introduction

It is an obvious fact that cancer cell lines bear only a partial resemblance to their origin. This issue has been addressed many times [1–6]; however, thus far, no practical guidelines on how to address this incongruity have been proposed, despite the fact that the basic and preclinical research is to a high extent based on cell line analysis (mostly due to the lack of a better model for a large scale use). We have already described the discrepancies in the *TP53* mutational profile between surgical samples and cell lines [7]. Moreover, we have recently reported differences in the status of several tumor suppressor genes (TSGs) between glioma cancer cell lines and surgical specimens [8]. Since a better model is not available, recognizing such differences might facilitate the adequate result interpretation, and thus, may be useful in various research projects from comparative biology studies to in vitro drug testing. Therefore, we decided to perform a comparison of in vivo and in vitro mutational profiles for other tumor suppressor genes. This would be the next step in understanding the discrepancies between the surgical samples and cell lines both in the frequency of homo- and heterozygous mutations and in their effect on protein sequence and structure.

Currently, the foundations of oncology are based on Knudson hypothesis (two-hit hypothesis), according to which tumorigenesis requires the elimination of both alleles of given tumor suppressor gene [9]. However, this model does not recognize the influence of the first allele elimination on cell biology. The exceptions to this pattern, such as the single-heterozygous mutations of *TP53*, *APC*, or *PTEN* in tumor samples, are usually explained as a consequence of dominant-negative effect (DNE), gain of function (GOF), and haploinsufficiency. Recently, Berger et al. proposed an extension of the two hit model, the continuum/quasi-sufficiency model, suggesting that one allele elimination/mutation is sufficient to importantly influence cell biology and certain genes, such as *PTEN*, can be entirely eliminated only at the latest stages of carcinogenesis, since their action temporarily protects preneoplastic cells against senescence/apoptosis [10]. This extended model inspired our analyses, since we assumed that cancer cell lines represent the most advanced stages of carcinogenesis. Furthermore, various neoplastic cells require special conditions to survive in vitro, for example, those with *IDH1* mutation or *EGFRvIII* [6, 11]. All these premises encouraged us to inquire if (and why) the status of tumor suppressor genes differs between surgical tumor specimens and cell lines.

## Materials and methods

The mutation frequency and microsatellite instability (MSI) data was extracted from the Sanger Institute Catalog Of Somatic Mutations In Cancer (COSMIC) database (v61 release) which gathers information on somatic mutations taken from the literature and in-house sequencing in human tumor samples and tumor cell lines [12–15]. For each gene in the database, the frequency of normal and mutated samples was extracted. Moreover, the mutated samples were divided into groups according to mutation zygosity (homozygosity, heterozygosity) and type (Table 1). Two main mutation groups were distinguished: mutations modifying or partially abolishing the protein function (mostly missense mutations) and mutations completely abolishing the protein function (mostly nonsense mutations and whole gene deletions) (Table 1). Splicing site mutations were not classified into either group because of their usually unpredictable effect on protein structure and function. The analyses were performed for each gene with Fisher’s exact test for 2 × 2 contingency tables calculated with Matlab 2010b (Mathworks) and R 2.15.1 package. The analysis comprised the comparison of homo- versus heterozygous mutations and those partially versus completely abolishing the protein function mutations in all their possible combinations.

**Table 1:** Classification of mutation type on the basis of the effect on protein structure and function

Modified protein (mutations modifying or partially abolishing the protein function)	Complex, deletion in frame
	Complex, insertion in frame
	Deletion, in frame
	Insertion, in frame
	Substitution, missense
No protein (mutations completely abolishing the protein function)	Complex, frame shift
	Deletion, frame shift
	Insertion, frame shift
	No detectable mRNA/protein
	Substitution, nonsense
	Whole gene deletion

Splicing site mutations were not classified into any of group, because of unpredictable effect on protein structure and sequence

## Results

Six hundred seventy cancer-associated genes were analyzed in 142,961 samples (137,708 tumor samples and 5,253 cell lines). The analysis of the summarized data (Supplementary tables) revealed the significant discrepancies in the frequencies of different mutation types between the surgical samples and cell lines. In oncogenes, as expected, there are almost no mutations resulting in the lack of protein and the proportions of missense heterozygous and missense homozygous mutations are retained in cell lines (exemplary data in Table 2: oncogene). In TSG, we observed two distinct recurrent patterns: a significant increase in the rate of homozygous mutations with the retention of the missense/nonsense proportion in cell lines or a simultaneous increase of the proportion of both homozygous and nonsense mutations in cell lines. *TP53* is an exemplary gene following the first pattern. In tumor samples, the frequencies of heterozygous and homozygous mutations are comparable (both for missense and nonsense), while the homozygous mutations are dominant in cell lines (the missense/nonsense proportion was retained; data and results of the statistical analyses in Table 2: classical two hits). Moreover, *RB1*, *NF1*, *NOTCH1*, and *PTEN* presented similar changes. The second pattern may be exemplified by the *SMAD4* gene, for which the proportion of nonsense homozygous mutations significantly increases in cell lines, while that of other mutation types decreases. For *CDKN2A* and *APC*, the mutation proportions were initially (in tumor samples) strongly shifted towards nonsense homozygous; however, the increase of this mutational type with the subsequent decrease of the other types of mutations was statistically significant. *BRCA2*, *SOCS1*, *STK11*, *MSH6*, and *SMARCA4* followed such a pattern as well (Table 2: three hits). Furthermore, we observed a group of genes for which the proportion of nonsense homozygous mutations in tumor samples was similar or higher than in the previously mentioned genes in cell lines. No further increase of this proportion was observed in cell lines; however, this may imply that such genes follow a similar pattern, but the process is already advanced in tumor samples (Table 2: three hits/final stage). Finally, we observed several less commonly analyzed genes which may potentially be classified as following the second pattern; however, the number of described samples was not large enough for a reliable analysis (Table 2: three hits/small group).

**Table 2:** A comparison of subgroups of homo/heterozygous mutation occurrence (HO/HE) and occurrence of mutations resulting in modified protein or lack of protein (MS/NS) in human cancer cell lines and surgical samples

Group	Gene		Number of samples	%MS-HE	%MS-HO	%NS-HE	%NS-HO	MS-HE//NS-HO	NS-HE//NS-HO	MS-HE//MS-HO	MS-HE//NS-HE	MS-HO//NS-HO	MS-HO//NS-HE
Classical 2 hits	*TP53*	TS	401	43.9	36.2	10.2	9.7	*p* < 0.001	*p* < 0.001	*p* < 0.001	*p* > 0.1	*p* > 0.1	*p* < 0.001
		CL	566	9.2	68.9	2.5	19.4						
	*RB1*	TS	47	10.6	21.3	34.0	34.0	*p* = 0.003	*p* < 0.001	*p* = 0.007	*p* > 0.1	*p* > 0.1	*p* < 0.001
		CL	102	1.0	33.3	2.0	63.7						
	*NF1*	TS	96	22.9	14.6	32.3	30.2	*p* > 0.1	*p* = 0.013	*p* = 0.007	*p* > 0.1	*p* = 0.098	*p* < 0.001
		CL	40	12.5	42.5	7.5	37.5						
	*NOTCH1*	TS	326	46.9	2.5	50.0	0.6	*p* < 0.001	*p* < 0.001	*p* < 0.001	*p* > 0.1	*p* = 0.141	*p* < 0.001
		CL	67	34.3	2.5	29.9	17.9						
	*PTEN*	TS	270	23.0	20.7	24.8	31.5	*p* < 0.001	*p* < 0.001	*p* < 0.001	*p* > 0.1	*p* > 0.1	*p* < 0.001
		CL	184	4.3	37.0	3.3	55.4						
3 hits	*BRCA2*	TS	17	35.3	17.6	11.8	35.3	*p* = 0.042	*p* < 0.001	*p* = 0.028	*p* > 0.1	*p* = 0.047	*p* > 0.1
		CL	17	0.0	35.3	17.6	47.1						
	*SMAD4*	TS	173	12.7	52.6	9.8	24.9	*p* < 0.001	*p* = 0.091	*p* = 0.033	*p* > 0.1	*p* = 0.003	*p* > 0.1
		CL	105	2.9	43.8	4.8	48.6						
	*SOCS1*	TS	18	55.6	22.2	22.2	0.0	*p* = 0.003	*p* = 0.061	*p* = 0.074	*p* > 0.1	*p* > 0.1	*p* > 0.1
		CL	15	13.3	40.0	13.3	33.3						
	*STK11*	TS	87	36.8	24.1	20.7	18.4	*p* < 0.001	*p* < 0.001	*p* = 0.002	*p* > 0.1	*p* = 0.005	*p* = 0.005
		CL	61	4.9	24.6	1.6	68.9						
	*MSH6*	TS	26	53.8	11.5	30.8	3.8	*p* < 0.001	*p* < 0.001	*p* = 0.002	*p* > 0.1	*p* > 0.1	*p* = 0.004
		CL	20	5.0	35.0	0.0	60.0						
	*SMARCA4*	TS	13	38.5	46.2	7.7	7.7	*p* = 0.063	*p* > 0.1	*p* > 0.1	*p* > 0.1	*p* = 0.040	*p* > 0.1
		CL	44	22.7	29.5	2.3	45.5						
	*APC*	TS	199	8.5	9.5	27.6	54.3	*p* = 0.126	*p* < 0.001	*p* > 0.1	*p* > 0.1	*p* = 0.025	*p* > 0.1
		CL	65	4.6	3.1	9.2	83.1						
	*CDKN2A*	TS	1,813	3.6	61.7	1.8	32.8	*p* < 0.001	*p* < 0.001	*p* < 0.001	*p* > 0.1	*p* < 0.001	*p* < 0.001
		CL	9,900	0.3	46.6	0.1	53.0						
3 hits/final stage	*BAP1*	TS	32	3.1	40.6	6.3	50.0	*p* > 0.1	*p* = 0.019	*p* > 0.1	*p* > 0.1	*p* > 0.1	*p* = 0.054
		CL	9	0.0	33.3	44.4	22.2						
	*FAM123B*	TS	97	2.1	37.1	14.4	46.4	*p* > 0.1	*p* > 0.1	*p* > 0.1	*p* > 0.1	*p* > 0.1	*p* > 0.1
		CL	7	14.3	14.3	0.0	71.4						
	*NF2*	TS	247	2.8	17.0	0.8	79.4	*p* > 0.1	*p* > 0.1	*p* > 0.1	*p* > 0.1	*p* = 0.001	*p* > 0.1
		CL	25	4.0	32.0	0.0	64.0						
	*SMARCB1*	TS	179	0.6	16.2	0.6	82.7	*p* > 0.1	*p* > 0.1	*p* > 0.1	*p* > 0.1	*p* > 0.1	*p* > 0.1
		CL	20	5.0	15.0	0.0	80.0						
	*VHL*	TS	415	4.6	39.0	2.9	53.5	*p* = 0.091	*p* = 0.036	*p* > 0.1	*p* > 0.1	*p* > 0.1	0.073
		CL	26	11.5	38.5	11.5	38.5						
3 hits/small group	*CDH10*	TS	6	83.3	16.7	0.0	0.0	*p* > 0.1	N/A	*p* > 0.1	*p* > 0.1	*p* > 0.1	*p* > 0.1
		CL	7	28.6	28.6	14.3	28.6						
	*MLH1*	TS	9	44.4	22.2	0.0	33.3	*p* > 0.1	*p* > 0.1	*p* > 0.1	*p* > 0.1	*p* > 0.1	*p* > 0.1
		CL	13	15.4	23.1	0.0	61.5						
	*MSH2*	TS	9	44.4	0.0	33.3	22.2	*p* > 0.1	*p* > 0.1	*p* = 0.076	*p* > 0.1	*p* > 0.1	*p* = 0.004
		CL	15	13.3	26.7	13.3	46.7						
	*SETD2*	TS	5	40.0	40.0	0.0	20.0	*p* > 0.1	*p* > 0.1	*p* > 0.1	*p* > 0.1	*p* > 0.1	*p* > 0.1
		CL	10	20.0	30.0	10.0	40.0						
Oncogene	*EGFR*	TS	768	81.6	17.8	0.3	0.3	*p* > 0.1	N/A	*p* > 0.1	N/A	N/A	N/A
		CL	26	84.6	15.4	0.0	0.0						
	*KIT*	TS	846	77.3	21.9	0.8	0.0	*p* > 0.1	N/A	*p* > 0.1	N/A	N/A	N/A
		CL	13	61.5	38.5	0.0	0.0						
	*KRAS*	TS	1,601	58.5	41.3	0.1	0.0	*p* > 0.1	N/A	*p* > 0.1	N/A	N/A	N/A
		CL	177	58.8	41.2	0.0	0.0						

Classical 2 hits, the genes which followed the 2-hit pattern; 3 hits, the genes which followed the 3-hit pattern; 3 hits/final stage, the genes which may have followed the 3-hit pattern, but the proportion of homozygous nonsense mutations is very high in tumors; 3 hits/small group, the genes which may have followed the 3-hit pattern, but the groups were not numerous enough to verify that

*TS* tumor sample, *CL* cell line, *MS* mutation resulting in modified protein, *NS* mutation resulting in lack of protein, *HO* homozygous mutation, *HE* heterozygous mutation

Since the number of cases with the reported status of zygosity was relatively low, we performed an additional analysis of the proportion of homo-/heterozygous and missense/nonsense mutations separately to verify the observed discrepancies in more numerous groups. In most genes, the results of both analyses were concordant (data in Table 3). Two of the genes initially classified as following the first pattern (*PTEN* and *RB1*) showed a statistically significant increase in the proportion of nonsense mutations in cell lines in comparison to tumor samples. On the other hand, for two genes classified as following the second pattern, (*SOCS1* and *MSH6*) the increase of the proportion of nonsense mutations in cell lines was not observed. In the genes potentially following the second pattern, the analysis of wider groups implies the increase in the proportion of homozygous and nonsense mutations; however, the statistical analyses do not confirm its significance.

**Table 3:** A comparison of homo/heterozygous mutation occurrence (HO/HE) and occurrence of mutations resulting in modified protein or lack of protein (MS/NS) in human cancer cell lines and surgical samples

Group	Gene	M tumor	% M tumor	M line	% M line	*p* value M	HO + HE tumor	% HO tumor	HO + HE line	% HO line	*p* value HO/HE	MS + NS tumor	% MS tumor	MS + NS line	% MS line	*p* value MS/NS
Classical 2 hits	*TP53*	14,015	75.03	681	65.36	*p* < 0.001	419	46.30	567	88.71	*p* < 0.001	13,071	81.99	677	79.32	*p* = 0.082
	*RB1*	214	7.00	151	14.80	*p* < 0.001	56	50.00	122	98.36	*p* < 0.001	167	19.16	121	9.09	*p* = 0.019
	*NF1*	320	14.95	39	4.56	*p* < 0.001	104	56.73	39	84.62	*p* = 0.002	283	22.97	40	22.50	*p* > 0.1
	*NOTCH1*	926	13.85	90	8.47	*p* < 0.001	295	15.93	55	34.55	*p* = 0.002	894	59.51	91	57.14	*p* > 0.1
	*PTEN*	1,793	11.50	395	18.68	*p* < 0.001	281	54.09	186	94.09	*p* < 0.001	1,626	42.19	369	30.08	*p* < 0.001
3 hits	*BRCA2*	50	2.55	16	1.85	*p* > 0.1	17	52.94	16	68.75	*p* > 0.1	46	52.17	18	33.33	*p* > 0.1
	*SMAD4*	251	8.11	124	10.39	*p* = 0.022	182	80.22	110	92.73	*p* = 0.004	243	44.03	115	20.00	*p* < 0.001
	*SOCS1*	38	3.95	17	1.96	*p* = 0.014	18	27.78	17	76.47	*p* = 0.007	40	55	16	56.25	*p* > 0.1
	*STK11*	200	5.41	69	6.51	*p* > 0.1	96	41.67	60	93.33	*p* < 0.001	181	51.38	64	18.75	*p* < 0.001
	*MSH6*	122	7.11	19	1.99	*p* < 0.001	26	26.92	28	77.78	*p* = 0.002	109	27.52	23	39.13	*p* > 0.1
	*SMARCA4*	36	7.86	50	4.66	*p* = 0.015	13	61.54	50	76.00	*p* > 0.1	35	91.43	44	36.36	*p* < 0.001
	*APC*	2,121	20.20	139	11.69	*p* < 0.001	205	65.85	75	88.00	*p* < 0.001	2,074	8.00	94	5.32	*p* > 0.1
	*CDKN2A*	2,513	12.29	1,172	38.45	*p* < 0.001	1,848	94.59	1,014	99.61	*p* < 0.001	2,369	17.56	966	10.14	*p* < 0.001
3 hits/final stage	*BAP1*	99	10.44	10	13.89	*p* > 0.1	33	90.91	9	55.56	*p* = 0.028	91	26.37	10	30.00	*p* > 0.1
	*FAM123B*	114	8.76	8	0.99	*p* < 0.001	98	83.67	8	75.00	*p* > 0.1	112	7.14	7	28.57	*p* > 0.1
	*NF2*	506	23.94	35	3.56	*p* < 0.001	277	96.75	33	93.94	*p* > 0.1	447	9.62	24	4.17	*p* > 0.1
	*SMARCB1*	240	19.67	44	16.18	*p* > 0.1	184	98.91	23	95.65	*p* > 0.1	230	13.04	35	17.14	*p* > 0.1
	*VHL*	1,123	22.44	90	8.65	*p* < 0.001	441	92.52	30	76.67	*p* = 0.009	1,018	40.57	85	45.88	*p* > 0.1
3 hits/small group	*CDH10*	13	6.16	5	10.2	*p* > 0.1	6	33.33	5	40.00	*p* > 0.1	10	100	7	71.43	*p* > 0.1
	*MLH1*	52	3.35	19	2.17	*p* > 0.1	13	61.54	19	89.47	*p* = 0.091	43	60.47	14	35.71	*p* > 0.1
	*MSH2*	37	3.26	15	1.84	*p* = 0.064	11	36.36	15	80	*p* = 0.043	29	34.48	15	13.33	*p* > 0.1
	*SETD2*	16	9.94	14	1.75	*p* < 0.001	6	50.00	14	57.14	*p* > 0.1	13	53.85	10	40.00	*p* > 0.1
Oncogene	*EGFR*	11,257	22.41	113	6.43	*p* < 0.001	793	18.16	29	17.24	*p* > 0.1	11,219	95.73	84	85.71	*p* < 0.001
	*KIT*	5,986	27.81	50	4.14	*p* < 0.001	1,053	18.33	15	33.33	*p* > 0.1	5,368	99.61	29	100	*p* > 0.1
	*KRAS*	21,938	22.89	631	18.34	*p* < 0.001	1,604	41.40	205	41.46	*p* > 0.1	21,806	99.99	394	100	*p* > 0.1

Classical 2 hits, the genes which followed the 2-hit pattern; 3 hits, the genes which followed the 3-hit pattern; 3 hits/final stage, the genes which may have followed the 3-hit pattern, but the proportion of homozygous nonsense mutations is very high in tumors; 3 hits/small group, the genes which may have followed the 3-hit pattern, but the groups were not numerous enough to verify that

*M* mutated sample, *MS* mutation resulting in modified protein, *NS* mutation resulting in lack of protein, *HO* homozygous mutation, *HE* heterozygous mutation

The analysis of microsatellite data from 810 cell lines and 720 primary samples revealed that the frequency of MSI was moderately higher in the former. High-frequency MSI (MSI-H) was detected in 66 cell lines and in 36 primary samples (8 vs. 5 %, *p* = 0,014). Any MSI (including high- and low-frequency MSI-L) was detected in 106 cell lines and in 69 primary samples (13 vs. 8.5 %, *p* = 0.036) (Table 4).

**Table 4:** A comparison of microsatellite instability frequency in human cancer cell lines and surgical samples. Fisher’s exact test results

	MSS	MSI		
		MSI-H	MSI-L	Total
Tumor	651	36	33	69
Line	704	66	40	106
	MSI-H vs. others	*p* = 0.01384	Any MSI vs. MSS	*p* = 0.03624

*MSS* microsatellite stability, *MSI* microsatellite instability, *MSI-H* high-frequency microsatellite instability (detected in at least 2 markers), *MSI-L* low-frequency microsatellite instability (detected in 1 marker)

## Discussion

In general, it is well known that cancer cell lines cannot be seen as a direct representation of the tumors; however, the extent of the differences between them may be underestimated. A deep analysis of mutation databases offers insight into this issue from another perspective. The presented study showed that homozygous mutations of many tumor suppressor genes are significantly more frequent in cell lines than in tumor samples. Similarly, nonsense mutations of such genes occur more frequently in vitro than in vivo. The quasi-sufficiency hypothesis proposed by Berger et al. offers a partial explanation for these discrepancies [9] (justifying the higher incidence of single heterozygous mutations in tumor samples, but not the preference of cell lines towards nonsense mutations). *PTEN* is an exemplary gene with such characteristics. Preneoplastic cells require a heterozygous mutation of this gene (with its function partially retained) during the early stages of carcinogenesis, as the cells without PTEN activity become senescent or die. Apparently, the hyperactivation of the PI3K pathway may be unfavorable at the early stages of carcinogenesis [16]. Most authors suggest that the complete *PTEN* elimination requires a prior neutralization of the genes required for oncogene-induced senescence (e.g., *TP53*, *CDKN2A*) [17–19].

**Fig. 1:**
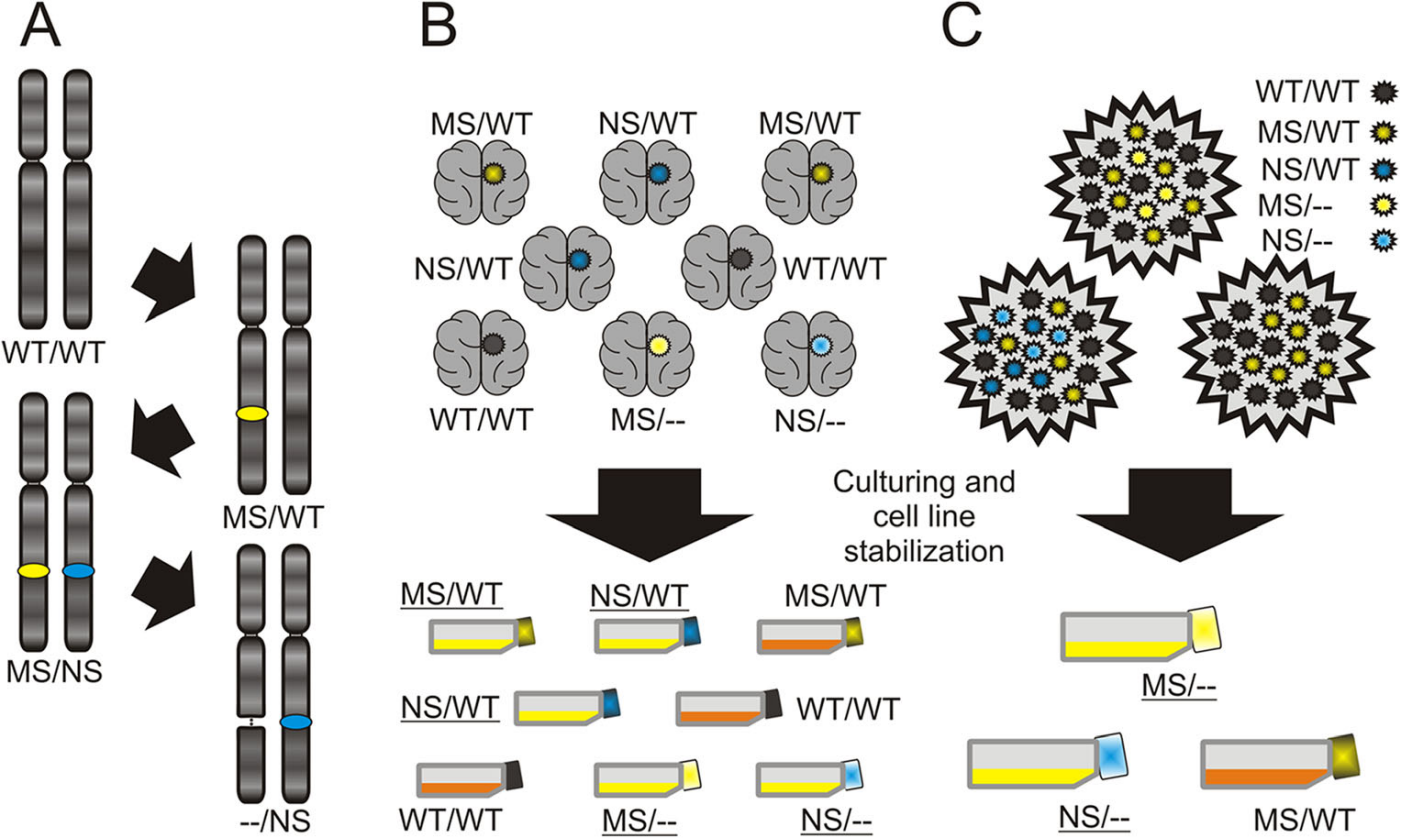
The hypotheses potentially explaining the differences in mutational profiles. **a** In vivo transformation of missense into nonsense mutations; this process may occur through various mechanisms, for example, a missense mutation of one allele is followed by a nonsense mutation of the other alleles and next by a deletion of the allele with the missense mutation. Missense mutations are marked as *yellow spots*, nonsense mutations as *blue* ones. **b** In vitro selection of tumor specimens which may give rise to stabilized cell lines. Obviously, the mutational status of a single gene may not determine the stabilization efficiency; however, the cumulative influence of all mutations may affect the probability of the successful cell line stabilization. The *color* of the tumor represents the mutation type of given gene (legend in the *top right-hand corner*), which is further reflected by the *color of the cap* of the respective culture flask. Stabilized cell lines are marked as *yellow bottles* with *underlined labels*; the others are marked as *orange bottles* with *normal labels*. **c** In vitro selection of cells within a tumor specimen which may give rise to a stabilized cell line. Again, within a heterozygous tumor the cells with certain molecular profiles may be more predisposed to the stabilization as a cell line. The *color* of the cells within tumors represents the mutation type of given gene (legend in the *top right-hand corner*), which is further reflected by the *color of the cap* of the respective culture flask. Stabilized cell lines are marked as *yellow bottles* with *underlined labels*; the others are marked as *orange bottles* with *normal labels*

Both missense and nonsense mutations of the *PTEN* gene almost equally eliminate the phosphatase activity of the protein; thus, the gradual change from missense heterozygous to nonsense homozygous would not be expected in this case [20]. On the other hand, genes such as *BRCA2* and *SOCS1* show the complex differences between surgery samples and cell lines—a significantly higher frequency of homozygous and nonsense (i.e., resulting in the complete lack of protein) mutations in the latter (Table 2). For such genes, we propose the “three hit” model (Fig. 1a). At the initial stages of carcinogenesis, a missense TSG mutation is sufficient/optimal through altering (yet not completely eliminating) the protein function. Next, a nonsense mutation is generated within the other allele. Finally, the allele with the missense mutation is deleted, causing the lack of protein. Other forms of in vivo changes are also possible, e.g., the missense mutation may be directly changed into a nonsense one etc.

These data may also offer an alternative explanation, assuming that among the multiple independent carcinogenic pathways, these with nonsense mutations are more adaptable in vitro and allow for more efficient cell line stabilization. This may refer both to intratumoral heterogeneity and to differences between cases (Fig. 1b, c). Therefore, cell lines would represent only a subgroup of cells/cases observed in vivo. Here, the transformation into the more advanced tumor stages is not accompanied by the missense to nonsense change. Nonetheless, still, the nonsense mutations may hypothetically cause some biological changes associated with the ease and effectiveness of cell line stabilization, e.g., the cells isolated from tumors with nonsense mutations may be more proliferative. *CDKN2A* is an exemplary gene whose molecular characteristic supports the in vitro adaptation hypothesis.

These hypotheses may seem mutually exclusive; however, both may be partially responsible for the observed differences. Still, irrespective of the underlying mechanism, such discrepancies are an incentive to consider the respective genes as following the quasi-sufficiency hypothesis.

Finally, we inquired whether the defective DNA damage response and repair mechanisms might be responsible for the observed differences in mutational profiles. For that purpose, we compared the MSI detection rates in cell lines and primary samples. Although the differences were statistically significant, microsatellite instability may not be the sole explanation of the observed results, due to its generally low detection rates (Table 4). Nonetheless, it may be one of the aspects affecting the cell line stabilization effectiveness.

The database analysis provides an additional argument that average cells from a cell line are more advanced in tumorigenesis than average cells from the corresponding surgical sample, e.g., the frequencies of detected TSG mutations are much higher in cell lines; however, the more thorough analysis of cell lines has to be emphasized.

In conclusion, we report the preferential character of nonsense mutations of TSGs in the most advanced cancer cells. Apparently, during the early stages of tumorigenic transformation, the complete elimination of many TSG may be lethal or otherwise unfavorable and becomes possible only when the required cellular/molecular context is achieved. Our hypothesis of “three hits” is a modification of the “continuum” model by Berger et al. [10]. Clearly, the potential transformation of missense to nonsense mutations during carcinogenesis requires more data. Alternatively, the presented data may be explained by the hypothesis that cell lines originating from tumors/cells with nonsense mutations are more easily stabilized in comparison to those with missense mutations. The differences in the mutational characteristics of TSG between tumor samples and cell lines may indicate the lack of the appropriate in vitro representation of tumors in vivo, which is particularly important from the drug testing perspective.

This study was supported by the National Science Center Grant No. 2011/01/B/NZ4/07832. Calculations in this paper have been carried out within Upper Silesian Center for Scientific Computational Science and Engineering (POIG.02.03.01-24-099/13).

## Electronic supplementary material

Below is the link to the electronic supplementary material.

Supplementary Table 1 (XLS 3430 KB)

Supplementary Table 2 (XLS 453 KB)

Supplementary Table 3 (XLS 245 KB)

Supplementary Table 4 (XLS 216 KB)

Supplementary Table 5 (XLS 199 KB)

Supplementary Table 6 (XLS 58 KB)

Supplementary Table 7 (XLS 47 KB)

Supplementary Table 8 (XLS 35 KB)
